# Cystathionine Gamma-Lyase Regulates TNF-α-Mediated Injury Response in Human Colonic Epithelial Cells and Colonoids

**DOI:** 10.3390/antiox13091067

**Published:** 2024-08-31

**Authors:** Francisco Arroyo Almenas, Gábor Törő, Peter Szaniszlo, Manjit Maskey, Ketan K. Thanki, Walter A. Koltun, Gregory S. Yochum, Irina V. Pinchuk, Celia Chao, Mark R. Hellmich, Katalin Módis

**Affiliations:** 1 Department of Surgery, The University of Texas Medical Branch at Galveston, Galveston, TX 77555, USA; 2 Division of Colorectal Surgery, Valley Health System, Las Vegas, NV 89119, USA; 3 Department of Surgery, Division of Colon & Rectal Surgery, The Pennsylvania State University, Milton S. Hershey Medical Center, Hershey, PA 17033, USA; 4 Department of Biochemistry & Molecular Biology & Surgery, The Pennsylvania State University College of Medicine, Hershey, PA 17033, USA; 5 Department of Medicine, The Pennsylvania State University College of Medicine, Hershey, PA 16802, USA

**Keywords:** cystathionine gamma-lyase, hydrogen sulfide, TNF-α, inflammation, colonic epithelial cells, colonoids

## Abstract

Cystathionine gamma-lyase (CSE) and TNF-α are now recognized as key regulators of intestinal homeostasis, inflammation, and wound healing. In colonic epithelial cells, both molecules have been shown to influence a variety of biological processes, but the specific interactions between intracellular signaling pathways regulated by CSE and TNF-α are poorly understood. In the present study, we investigated these interactions in normal colonocytes and an organoid model of the healthy human colon using CSE-specific pharmacological inhibitors and siRNA-mediated transient gene silencing in analytical and functional assays in vitro. We demonstrated that CSE and TNF-α mutually regulated each other’s functions in colonic epithelial cells. TNF-α treatment stimulated CSE activity within minutes and upregulated CSE expression after 24 h, increasing endogenous CSE-derived H_2_S production. In turn, CSE activity promoted TNF-α-induced NF-ĸB and ERK1/2 activation but did not affect the p38 MAPK signaling pathway. Inhibition of CSE activity completely abolished the TNF-α-induced increase in transepithelial permeability and wound healing. Our data suggest that CSE activity may be essential for effective TNF-α-mediated intestinal injury response. Furthermore, CSE regulation of TNF-α-controlled intracellular signaling pathways could provide new therapeutic targets in diseases of the colon associated with impaired epithelial wound healing.

## 1. Introduction

Under physiological conditions, the mucosal surface of the colon is covered with epithelial cells that form a barrier to protect the host from luminal bacteria, toxins, pathogenic antigens, and other harmful substances [1,2]. Loss of epithelial integrity may occur due to infection, chemical or physical insults, ischemia, irradiation, or chronic inflammation [1]. Normally, precisely timed and regulated cellular mechanisms are in place to initiate and execute the process of colonic epithelial wound healing in response to injury to reestablish properly functioning colonic mucosa [2,3]. Disruption of these homeostatic processes results in pathologies such as different forms of inflammatory bowel disease (IBD) characterized by recurrent or chronic impairment of mucosal barrier integrity, bacterial infiltration into the bowel wall, pronounced inflammation, and compromised wound healing [4,5,6,7]. Elucidating the physiological regulation of intestinal injury response and identifying specific factors that improve wound healing in the colon could contribute to new therapeutic strategies in IBD and other intestinal diseases.

Tumor necrosis factor-alpha (TNF-α) has been shown to exert a broad scope of homeostatic and pathogenic bioactivities in the gut, including intestinal inflammation and wound healing [8,9,10]. It is mainly secreted by immune cells and impacts different functions of multiple cell types in the wound microenvironment, including epithelial cells [11]. Under homeostatic conditions, TNF-α not only drives the inflammatory responses but also plays a central role in restoring mucosal integrity in response to injury [12,13]. During mucosal healing, TNF-α orchestrates a multi-step process to facilitate wound closure [14,15]. Thus, a well-regulated TNF-α activity is necessary for physiological wound healing and maintaining epithelial barrier function. Conversely, under pathological conditions, such as IBD, the TNF-α level rises in an uncontrolled manner, causing barrier integrity disruption and mass death of colonocytes, leading to epithelial injury, bacterial and immune cell infiltration of the intestinal wall, and chronic inflammation [16,17]. Even though several neutralizing antibodies against TNF-α have been recently approved for treating ulcerative colitis (UC), one of the major forms of IBD, their effectiveness in sustaining remission of the disease is only 17–40% [17,18,19,20], suggesting that the role and regulation of TNF-α signaling in colonic inflammation and wound healing is still poorly understood. TNF-α signals through two distinct cell membrane receptors, TNF receptor 1 and 2 (TNFR1 and TNFR2), in a complex and tightly regulated process that involves a multitude of intracellular signal transductor molecules and protein complexes arranged into interacting signaling cascades [9,21,22]. Several research groups have demonstrated that some of the key effector molecules of these signaling pathways, including nuclear factor kappa-B (NF-κB), as well as mitogen-activated protein kinases (MAPKs), p38, and extracellular signal-regulated kinase 1/2 (ERK1/2), play a fundamental role in TNF-α signaling of colonic epithelial cells [23,24,25,26]. In fact, MAPK and NF-κB activity has been shown to be indispensable for epithelial homeostasis and wound healing [26,27,28].

The gaseous signaling molecule hydrogen sulfide (H_2_S) has emerged as an important mediator of gastrointestinal homeostasis, maintaining mucosal integrity, limiting inflammation, and promoting wound healing in response to epithelial injury [29,30,31]. A major source of endogenous H_2_S production in colonic epithelial cells is the trans-sulfuration pathway, in which the key role of cystathionine gamma-lyase (CSE) has been demonstrated, but the relative contribution of CSE and other H_2_S sources (i.e., cystathionine β-synthase (CBS), 3-mercaptopyruvate-sulfurtransferase (3-MST), selenium-binding protein 1, and D-amino acid oxidase) to the overall H_2_S production under homeostatic and inflammatory conditions are mostly unknown and highly debated [32,33,34,35,36,37]. We and others have demonstrated that the inhibition of CSE- or CBS-dependent H_2_S production in animal models of colitis exacerbated inflammation and delayed regeneration, while the administration of H_2_S donors increased resistance to injury and accelerated tissue repair [36,38,39,40,41]. Our recent report of decreased CSE expression in colon epithelial tissue samples of inflamed UC specimens strongly implicated the critical role of this enzyme in the pathophysiology of chronic inflammation in the colon [41,42]. Accumulating studies in the pathomechanism of colitis suggest that H_2_S also plays a key role in suppressing aggravated immune responses [43], regulating NF-κB activation and cytokine signaling [44,45], as well as modulating barrier function via altered status of epithelial tight junctions [46]. Most currently available data about the biological role of H_2_S and its producing enzymes in the colon epithelium were obtained from pathological conditions, while there is a paucity of information regarding their function in colonocytes under physiological conditions.

To date, the mechanism of how the CSE/H_2_S axis regulates normal intestinal wound healing remains to be thoroughly defined. Since there is considerable overlap between the biological processes regulated by endogenous H_2_S and TNF in the colon, and low CSE levels correlate with dysregulated TNF-α signaling in UC, we hypothesized that they share a common mechanism. In the current study, we have shown that in colonic epithelial cells, TNF-α upregulates H_2_S production, as well as CSE expression and activity. We have also found that CSE is responsible for most of the increased early H_2_S production induced by TNF-α, and CSE activity regulates some, but not all, signal transduction pathways orchestrated by TNF-α during injury response. We have identified TNF-α-induced NF-κB and ERK1/2 activation, but not p38 MAPK phosphorylation, to be regulated by CSE. Finally, we have demonstrated that CSE activity is required for the physiological processes restoring colonic epithelial integrity after injury. In summary, our results suggest that the endogenous CSE/H_2_S axis regulates TNF-α signaling, plays a vital role in maintaining intestinal barrier integrity, and decreased CSE activity could lead to impaired epithelial wound healing.

## 2. Materials and Methods

### 2.1. Materials

Dulbecco’s modified Eagle’s medium (DMEM), phosphate-buffered saline (PBS), Bovine Serum Albumin (BSA), DL-propargylglycine (PAG), ß-cyanoalanine (BCA), hydrocortisone, insulin, transferrin, gentamicin sulfate, and sodium selenite were obtained from MilliporeSigma (St. Louis, MO, USA). MAPK inhibitors PD98059 and SB203580 were purchased from Bio-Techne Corp (Minneapolis, MN, USA). Recombinant human TNF-α protein was purchased from R&D Systems (Minneapolis, MN, USA). The slow-releasing H_2_S donor molecule, GYY4137, was purchased from Cayman (Ann Arbor, MI, USA). Epidermal growth factor (EGF) was procured from PeproTech (Cranbury, NJ, USA). Cosmic Calf Serum was obtained from GE Healthcare Life Sciences (Hyclone, GE Healthcare Life Sciences, Chicago, IL, USA). For organoid cultures, IntestiCult™ Organoid Growth Medium, IntestiCult™ Organoid Differentiation Medium, Gentle Cell Dissociation Reagent (all STEMCELL Technologies Inc., Cambridge, MA, USA), DMEM/F12 medium (Gibco, Thermo Fisher Scientific, Waltham, MA, USA), and Matrigel (Corning, Glendale, AZ, USA) were used. Western blot antibodies were obtained from Proteintech Group Inc. (Rosemont, IL, USA), MilliporeSigma, and Cell Signaling Technology (Danvers, MA, USA). Protease and phosphatase inhibitor cocktail, dithiothreitol (DTT), phenylmethylsulfonyl fluoride (PMSF), NaCl, EDTA, EGTA, HEPES, sucrose, NP40, Pierce bicinchoninic acid protein assay kit, and enhanced chemiluminescent (ECL) HRP substrate for Western blot analysis were purchased from Thermo Fisher.

### 2.2. Cell Culture

The immortalized human colonic epithelial cell (HCEC) line was provided by Dr. Shay’s laboratory (Jerry Shay Ph.D., University of Texas Southwestern Medical Center, Dallas, TX, USA) and maintained in DMEM supplemented with additives, as described previously [47]. The HCEC 1CT cells (referred to as HCEC throughout the manuscript) were established in Dr. Shay’s laboratory as a diploid epithelial cell line derived from non-malignant, normal colonic tissue biopsied from a patient undergoing routine colonoscopy screening. Cells were immortalized by expressing the non-oncogenic proteins cyclin-dependent kinase 4 (Cdk4) and the catalytic component of human telomerase (hTERT). HCECs have been shown to exhibit morphological features and markers characteristic of normal colonic epithelial cells. The cells were grown in high-glucose Dulbecco’s modified Eagle medium supplemented with 2% Cosmic Calf Serum, EGF (25 ng/mL), hydrocortisone (1 μg/mL), insulin (10 μg/mL), transferrin (2 μg/mL), sodium selenite (5 nM), and gentamicin sulfate (50 μg/mL). HCECs were cultured in flasks or dishes with unique attachment surfaces (Primaria^®^ BD Biosciences, San Jose, CA, USA) and kept under a 2% oxygen and 5% carbon dioxide atmosphere at 37 °C to maintain a stable normal karyotype (46, XY). Cell morphology was closely monitored to ensure the biological relevance of the experimental results.

### 2.3. Organoid Culture

Healthy human colon-derived organoids (colonoids) were provided by Dr. Yochum’s laboratory (Gregory S. Yochum, Ph.D., Penn State University College of Medicine, Hershey, PA, USA) from the Carlino Family Inflammatory Bowel and Colorectal Disease Biobank (Milton S. Hershey Medical Center, Hershey, PA, USA). Colonoids were cultured in IntestiCult™ Organoid Growth Medium or IntestiCult™ Organoid Differentiation Medium (STEMCELL Technologies) following the manufacturer’s protocol. Briefly, colonoids (suspended in DMEM/F12 medium containing 1% BSA) were seeded as 50 μL domes of Matrigel/colonoid suspension (50% mixture) in pre-warmed (37 °C), 24-well tissue culture plates, with one dome/well. The plates were placed in a tissue culture incubator (37 °C, 5% CO_2_) for 20 min to let the domes solidify, then 750 μL of pre-warmed (37 °C) organoid growth medium was added to each well, and the colonoid cultures were kept in a tissue culture incubator (37 °C, 5% CO_2_) for 7 days with growth medium changes every 3 days. For expansion, after 7 days of growth, the organoids were mechanically disrupted and suspended in Gentle Cell Dissociation Reagent (STEMCELL Technologies). Single cells and small cell clusters were centrifuged, and the pellets were resuspended in DMEM/F12 medium containing 1% BSA, then seeded as 50 μL domes of Matrigel/colonoid suspension as before, at a passage ratio of 1:2–1:4. For experiments, the growth medium was switched to a differentiation medium on day 4 after seeding and maintained for 2 additional days before treatment with inhibitors and/or TNF-α.

### 2.4. Western Blot Analysis

Cells were lysed in Nonidet P-40 buffer (50 mmol/L Tris-HCl pH 8.0, 150 mmol/L NaCl, 1% Nonidet P-40) supplemented with protease and phosphatase inhibitors, diluted in NuPAGE LDS Sample Buffer (Thermo Fisher), and boiled at 95 °C. Lysates (20–40 µg protein/10 µL/well) were resolved on 4–12% NuPage Bis-Tris acrylamide gels (Thermo Fisher) and transferred to PVDF membranes. Membranes were blocked with Starting Block T20 (Thermo Fisher) and then were probed overnight at 4 °C with primary antibodies anti-CSE (Proteintech), anti-phospho-ERK1/2, or anti-phospho-p38 (Cell Signaling) at a 1:1000 dilution in blocking buffer. The corresponding anti-rabbit (Cell Signaling) or anti-mouse (MilliporeSigma) horseradish peroxidase-conjugated secondary antibodies were used at 1:3000 dilution, at RT for 90 min. To normalize signals, the membranes were re-probed with anti-β-actin (MilliporeSigma), GAPDH, or β-tubulin (both from Proteintech) antibodies at 1:3000 dilution. Enhanced chemiluminescent substrate (Pierce Biotechnology, Thermo Fisher) was used to detect the signal in a camera-based chemiluminescence detection system (GBox, Syngene USA, Frederick, MD, USA). The intensity of Western blot signals was quantified by densitometry using the ImageJ 1.45s software (National Institutes of Health, Bethesda, MD, USA). The ratios of the signals were expressed as normalized densitometry units.

### 2.5. Small-Interfering RNA (siRNA)-Mediated CSE Silencing

SiRNA oligonucleotides (Silencer Select Pre-designed siRNAs #s3710, Thermo Fisher) were used for transient silencing of CSE in HCECs, as described earlier [48]. To optimize attenuation efficiency, preliminary transfections were carried out at 10–100 nM siRNA concentrations. For the negative control, commercially available, non-targeting siRNAs (Silencer Select Negative Control #1 siRNA, Thermo Fisher) were used in an identical manner to the optimized silencing protocol. Briefly, HCEC cells were seeded into 6-well Primaria^®^ plates (BD Biosciences). The following day, the growth medium was replaced with Opti-Mem medium lacking antibiotics, followed by transfection with siRNA fragments (20 nM final concentration) and Lipofectamine RNAiMAX complexes (all Thermo Fisher). Control cells were transfected in parallel with non-targeting siRNA. After 20 h, the transfection medium was replaced by fresh growth medium. Experiments were performed 48 h after transfection. Silencing efficiency of CSE was assessed by Western blot densitometric analysis (Figure S1).

### 2.6. NF-ĸB Translocation Assay

HCEC cells were cultured on type 1 collagen-covered coverslips until they reached confluency. The cells were pretreated for an hour with the CSE inhibitor PAG or BCA (3.0 mM), the ERK1/2 MAPK inhibitor PD98059 (30 μM), or the p38 MAPK inhibitor SB203580 (10 μM), followed by the addition of TNF-α (10 ng/mL) or the vehicle control to the respective groups for 30 min at 37 °C. The cells were then washed twice with ice-cold PBS, fixed with 4% paraformaldehyde for 15 min, permeabilized with 0.3% Triton X-100 for 10 min, and blocked with 1% BSA containing phosphate-buffered saline (PBS) at RT for 20 min. The permeabilized cells were incubated with the primary anti-NF-ĸB p65 antibody (1:100 dilution; Santa Cruz Biotechnology Inc., Dallas, TX, USA) overnight at 4 °C. The following day, all coverslips were washed three times with PBS and incubated with a goat anti-rabbit IgG antibody labeled with Alexa 488, and cell nuclei were labeled by DAPI staining (both Thermo Fisher). The specific immunostaining was visualized using immunofluorescence microscopy (Zeiss Axio Imager M2 microscope system, Jena, Germany), and image analysis was performed using ImageJ 1.45s software (National Institutes of Health, Bethesda, MD, USA). Corrected total cell fluorescence (CTCF) was calculated using the formula: CTCF = integrated fluorescence density − (area of selected cell × mean fluorescence of background readings). An average of 25 representative nuclei was analyzed per coverslip (at least 3 coverslips per treatment group).

### 2.7. Separation of Cytoplasmic and Nuclear Extracts

Cytoplasmic and nuclear fractionation was performed as described previously [49], with minor modifications. Briefly, after experiments, HCEC monolayers were washed with cold PBS, then scraped into cold PBS freshly supplemented with 1 mM DTT and 0.5 mM PMSF. Collected cell scrapings were centrifuged at 1500× *g* for 5 min at 4 °C, then cell pellets were resuspended with cold Buffer A (10 mM HEPES-pH 7.4, 10 mM NaCl, 0.1 mM EDTA, 0.1 mM EGTA, 1× protease and phosphatase inhibitor cocktail, 1 mM DTT, and 0.5 mM PMSF) and incubated on ice for 15 min. NP40 was added to a final concentration of 0.5% and the tubes containing the cell pellets were vortexed for 30 s then incubated on ice for 3 min. After centrifugation at 4000× *g* for 2 min at 4 °C, the supernatant was saved as the cytoplasmic fraction and the pellet was further processed to yield the nuclear fraction.

The pellet was resuspended in ice-cold Buffer B (1.7M sucrose, 10 mM HEPES-pH 7.4, 10 mM NaCl; 0.1 mM EDTA; 0.1 mM EGTA, 1× protease and phosphatase inhibitor cocktail, 1 mM DTT, and 0.5 mM PMSF), then centrifuged for 30 min at 15,000× *g* at 4 °C. The resultant nuclear pellets were resuspended in ice-cold Buffer C (20 mM HEPES, 0.4 M NaCl, 1 mM EDTA, 1 mM EGTA, 1× protease and phosphatase inhibitor cocktail, 1 mM DTT, and 0.5 mM PMSF) and placed in a shaker for 1 h at 4 °C. After centrifugation at 15,000× *g* for 5 min at 4 °C, the supernatant was saved for the nuclear extract. Cytoplasmic and nuclear extracts of each sample were normalized for protein amounts using the Pierce BCA protein assay kit (Thermo Fisher). Successful fractionation was verified by Western blotting of cytoplasmic and nuclear extracts using antibodies against cytoplasmic (β-tubulin) and nuclear (histone deacetylase 1 (HDAC1)) housekeeping proteins (Figure S2).

### 2.8. Measuring H_2_S Production in Live Cells

The 7-azido-4-methylcoumarin (AzMC, MilliporeSigma) is a cell-membrane-permeable fluorogenic probe, used to detect the generation of H_2_S in intact cells. The aromatic azide moiety is selectively reduced in the presence of H_2_S, producing the fluorescence agent with a concomitant increase in the fluorescence signal, with Ex = 365 nm and Em = 450 nm. The probe is highly sensitive and selective for H_2_S, providing a linear detection range in vitro between 200 nM and 100 μM H_2_S. To measure H_2_S production in live cells, HCECs were seeded in type 1 collagen-coated coverslips until they reached confluency. Before the assay, the cells were serum-starved for 48 h. First, the cells were loaded with 10 μM of AzMC for 30 min, as previously described [50]. Cells were then pretreated for an hour with either CSE inhibitors (PAG or BCA, 3 mM) or vehicle, followed by the addition of TNF-α (10 ng/mL). GYY4137 (400 μM) was used as a positive control of the assay to detect non-enzymatic H_2_S generation. Finally, the cells were washed with PBS, and the specific fluorescence signal was visualized and measured using fluorescence microscopy (Zeiss Axio Imager M2 microscope system with Stereo Investigator Software version 2022, Jena, Germany).

### 2.9. Assessing Transepithelial Electrical Resistance (TEER)

Transepithelial electrical resistance (TEER) is the measurement of electrical resistance across the epithelial cell monolayer—it is a sensitive and reliable method to confirm the integrity and permeability of the monolayer [51]. HCECs were grown in type 1 collagen-coated, 24-well ThinCert™ transwell plates (Greiner Bio-One, Monroe, NC, USA) for 6 days or until the TEER measured ~400 Ohms (W). Culture media was replaced with 1% Bovine Serum Albumin (BSA) containing starvation media for 2 h. Then, cultures were pretreated for an hour with either CSE inhibitors (PAG or BCA, 3 mM) or MAPK inhibitors (PD98059, 30 μM; SB203580, 10 μM), followed by the addition of TNF-α (10 ng/mL). TEER was measured using an EVOM2™ Voltohmmeter (World Precision Instruments, Sarasota, FL, USA) every 2 to 6 h over a 24-h-long period.

### 2.10. Wound Scratch Assay

HCECs were grown in type 1 collagen-coated 12-well plates. When cells reached confluency, the culture medium was replaced with 1% BSA containing starvation media for 48 h. Cells were pretreated for an hour with either CSE inhibitors (PAG or BCA, 3 mM) or MAPK inhibitors (PD98059, 30 μM; SB203580, 10 μM). Afterward, a vertical scratch wound was made in a straight line across the diameter of each well by using a 200 μL sterile pipette tip and a ruler. The cells were washed with PBS, and fresh media was added, followed by the addition of TNF-α (10 ng/mL). Images of the wound were taken right after initiation and then every 6 h until the scratched wounds completely disappeared by 48 h. The ratio of wound closure was determined by measuring the wound area at each time point and normalizing it to the initial wound size using ImageJ 1.45s software (National Institutes of Health).

### 2.11. Data and Statistical Analysis

Statistical analyses were performed using GraphPad Prism 9 analysis software (GraphPad Software Inc., La Jolla, CA, USA). Data are shown as mean ± SEM unless otherwise indicated. The normal distribution of individual datasets was determined using the Shapiro–Wilk and the D’Agostino and Pearson normality tests. Statistical analyses included the unpaired, nonparametric Mann–Whitney test, one-way ANOVA with Dunnett’s multiple comparisons, and two-way ANOVA followed by Tukey’s post hoc test to determine differences between experimental groups. Each experimental group or condition was repeated at least in three replicates and performed independently on at least three different experimental days. A value of *p* ≤ 0.05 was considered statistically significant. Figures were generated using GraphPad Prism 9 (GraphPad) and PowerPoint Version 2309 (Microsoft Corporation, Redmond, WA, USA) software. The graphical abstract of this manuscript was adapted from “TNF Pathway” using BioRender.com (2023) and retrieved from https://app.biorender.com/biorender-templates (accessed on 1 January 2023). 

## 3. Results

### 3.1. CSE Expression Is Elevated by TNF-α Stimulus in Colonocytes

**Figure 1:**
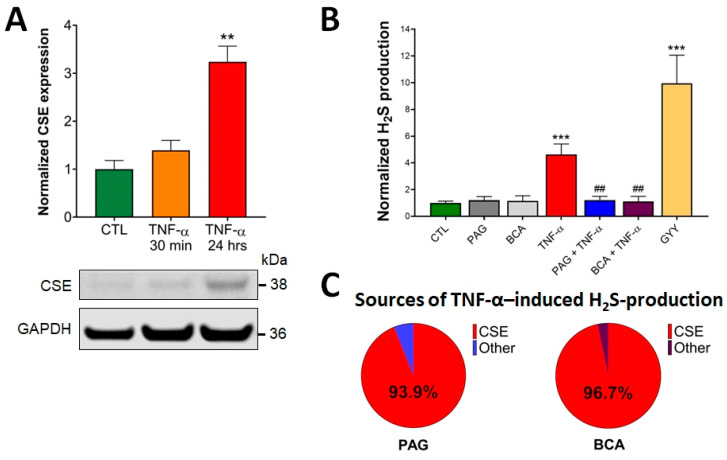
TNF-α upregulates CSE protein expression and CSE-derived H_2_S production. (**A**) Representative Western blot image shows the CSE protein expression level in control (CTL, mock-treated) and TNF-α-treated (10 ng/mL, 30 min or 24 h) HCECs. Densitometric analyses of 3 independent experiments revealed that a 24 h TNF-α treatment of HCECs resulted in an approximately 3-fold increase in CSE protein expression, while a 30 min treatment elicited no significant change. (**B**) H_2_S levels were determined in live HCECs using a cell-membrane-permeable H_2_S scavenging fluorescence probe (AzMC, 10 μM). The signal was visualized using fluorescence microscopy and expressed as relative fluorescence units (RFU). TNF-α (10 ng/mL, 30 min) significantly increased H_2_S production, which was inhibited by pretreatment with specific CSE inhibitors, PAG or BCA (3 mM, 1 h). Control cells (CTL) received the vehicle both times (pretreatment and treatment). H_2_S donor GYY4137 (400 μM, 30 min) was used as a positive control. (**C**) CSE-derived H_2_S production was determined by comparing H_2_S measurements with and without pretreating the cells with PAG or BCA. CSE was responsible for over 90% of all TNF-α-induced H_2_S production, as determined by PAG (93.9%) and BCA (96.7%). Data are shown as mean ± SEM of *n* ≥ 3 independent experiments. ** *p* ≤ 0.01 and *** *p* ≤ 0.001, significantly different from the control (CTL) group, and ^##^ *p* ≤ 0.01, significantly different from the TNF-α group: one-way ANOVA followed by Dunnett’s post hoc test.

First, we assessed the effects of TNF-α treatment on CSE expression and H_2_S production in HCECs. Western blot analysis of HCEC lysates demonstrated detectable CSE protein expression under basal conditions (CTL, untreated), which was significantly elevated by the addition of TNF-α (10 ng/mL) for 24 h (Figure 1A). This TNF-α concentration was chosen based on dose-dependent efficacy studies (in a range from 0.1 ng/mL to 1 mg/mL) in proliferation and barrier function assays, as well as cytotoxicity experiments (LDH assay). No cytotoxicity was detected up to 100 ng/mL, and the 10 ng/mL TNF-α concentration exerted maximum effect in both functional assays. A shorter, 30-min-long exposure time with TNF-α did not substantially alter CSE expression compared to basal conditions. These data suggest that any short-term CSE-mediated biological effects in response to TNF-α stimuli are caused by altered CSE enzymatic activity, independent of changes in the protein expression level. In contrast, any long-term reactions could be associated with significantly increased CSE protein expression, in addition to potentially altered CSE function.

### 3.2. CSE-Derived H_2_S Production Is Increased by TNF-α

To measure CSE-derived H_2_S production in live cells, HCEC cells were loaded with a fluorescent H_2_S probe (AzMC, 10 μM) for 30 min, as previously described [50,52]. HCECs were then treated with the pharmacological inhibitors of CSE (PAG or BCA, 3 mM) for an hour, followed by the addition of TNF-α (10 ng/mL). GYY4137 (400 μM), an H_2_S-releasing compound, was used as a positive control of the assay. We used these chemicals at concentrations determined to be efficient without causing any cellular toxicity in earlier studies [41]. The specific fluorescence signal was visualized using fluorescence microscopy, and normalized signal intensities under different treatment conditions were compared. We found that pretreating the cells with PAG or BCA alone did not alter the endogenous H_2_S production of HCECs at a detectable level compared to the untreated CTL group (Figure 1B). However, the addition of TNF-α significantly elevated intracellular H_2_S production, which was completely blocked by the administration of PAG or BCA. When we calculated the proportion of CSE-derived H_2_S production in the total TNF-α-induced H_2_S increase using the data obtained by the CSE inhibitors, we found that CSE was responsible for over 90% of it (Figure 1C). Together these findings confirmed that the activity of CSE plays a critical role in TNF-α-induced H_2_S production in colon epithelial cells.

### 3.3. CSE/H_2_S Axis Regulates TNF-α-Induced MAPK and NF-ĸB Signaling

**Figure 2:**
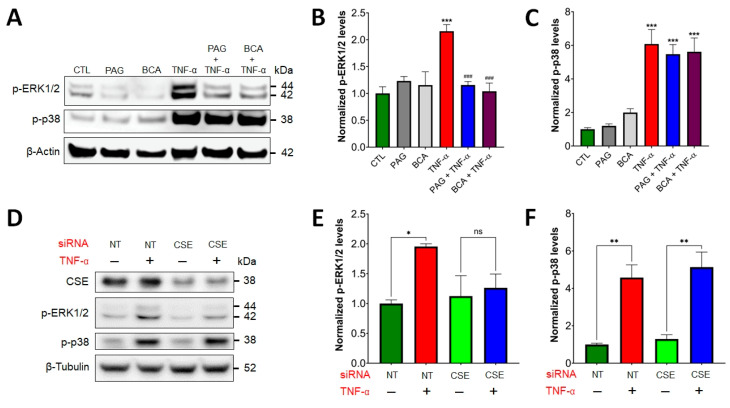
CSE regulates TNF-α-induced ERK1/2 signaling. (**A**–**C**) HCECs were pretreated with specific CSE inhibitors, PAG or BCA (3 mM, 1 h), or the vehicle control, then treated with TNF-α (10 ng/mL, 30 min) or mock-treated. Control cells (CTL) received the vehicle both times. ERK1/2 and p38 activation were analyzed by Western blotting using antibodies against phosphorylated ERK1/2 and p38, respectively. Representative Western blot images (**A**) and densitometric analyses of *n* ≥ 3 independent experiments (**B**,**C**) showed that TNF-α stimulated ERK1/2 and p38 activation. CSE blockage by PAG or BCA decreased TNF-α-mediated ERK1/2 activation, but it did not affect p-p38 levels. (**D**–**F**) HCECs were transfected with siRNAs targeting CSE mRNAs or non-target (NT) controls. Then, 48 h after transfection, the cells were treated with TNF-α (10 ng/mL, 30 min) or mock-treated. Representative Western blot images (**D**) and densitometric analyses of *n* ≥ 3 independent experiments (**E**,**F**) verified that silencing CSE expression abolished TNF-α-stimulated ERK1/2 activation, but it did not affect p-p38 levels. *** *p* ≤ 0.001, significantly different from the control (CTL) group, and ^###^ *p* ≤ 0.001, significantly different from the TNF-α group: one-way ANOVA followed by Dunnett’s post hoc test. *p ≤ 0.05 and **p ≤ 0.01 significantly different from control groups (NT and CSE), as well as “ns” indicates non-significant differences between two groups: unpaired Student’s *t*-test.

We examined the functional role of the CSE/H_2_S axis in three distinct TNF-α-activated pathways: ERK1/2 and p38 MAPK signaling, as well as the activation of NF-ĸB. Western blot analysis of HCEC lysates demonstrated rapid, TNF-α-stimulated phosphorylation of ERK1/2 and p38 MAPK (Figure 2). Intriguingly, after pretreating the cells with the pharmacological CSE inhibitors, PAG or BCA (3 mM), TNF-α-induced ERK1/2 activation was diminished (Figure 2A,B). In contrast, p38 activation by TNF-α was insensitive to CSE blockage (Figure 2A,C), indicating that some, but not all TNF-α-mediated signaling pathways are regulated by CSE.

To verify the presence or absence of regulatory effects by CSE on these two pathways in colonic epithelial cells, we transfected HCECs with a siRNA targeting CSE and confirmed silencing efficiency by Western blotting and densitometric analysis (Figure S1). For negative control, we used HCECs transfected with non-targeting siRNAs. Similar to our results with CSE inhibitors, we found that silencing CSE expression abolished TNF-α-stimulated ERK1/2 activation, but it did not affect p-p38 levels (Figure 2D–F).

**Figure 3:**
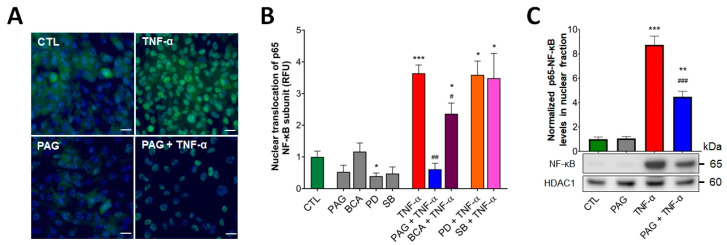
CSE regulates TNF-α-mediated NF-ĸB activation. (**A**,**B**) Nuclear translocation of p65 subunit of NF-ĸB was visualized in HCECs by immunofluorescence staining. The cells were pretreated with inhibitors PAG, BCA (both 3 mM), PD98059 (PD, 30 μM), SB203580 (SB, 10 μM), or vehicle for 1 h, then treated with TNF-α (10 ng/mL) or vehicle for 30 min. Control cells (CTL) received the vehicle both times. After the treatments, the cells were fixed, and p65 protein was labeled with Alexa 488. Nuclei were visualized by DAPI staining. Representative immunofluorescent images of coverslip cultures taken at 400× magnification (**A**) and normalized relative fluorescence intensity levels (RFU) of HCEC treatment groups (**B**) showed that TNF-α-stimulated NF-ĸB activation was reduced by CSE inhibitors (PAG and BCA) but remained unaffected by p-ERK1/2 and p-p38 inhibitors (PD and SB). (**C**) Effects of CSE inhibition on nuclear p65-NF-ĸB levels in HCECs after the same PAG/TNF-α treatments were verified by nuclear fractionation and Western blot analysis/densitometry. Data are shown as mean ± SEM of *n* ≥ 3 independent experiments. * *p* ≤ 0.05, ** *p* ≤ 0.01, or *** *p* ≤ 0.001, significantly different from the control (CTL) group; ^#^ *p* ≤ 0.05, ^##^ *p* ≤ 0.01, or ^###^ *p* ≤ 0.001, significantly different from the TNF-α group: unpaired, nonparametric Mann–Whitney test. Scale bars indicate 10 μm.

Under homeostatic conditions, proper regulation of NF-ĸB results in colonic epithelial restitution and tissue repair. We investigated whether TNF-α-induced NF-ĸB activation was regulated by CSE activity. Using an immunocytochemistry assay, we visualized the nuclear translocation of the p65 subunit of NF-ĸB as an indicator of NF-ĸB activation. HCEC cells were pretreated with the pharmacological inhibitors of CSE (PAG or BCA, 3 mM), ERK1/2 (PD98059, PD—a MEK1/2 inhibitor, commonly used to inhibit the phosphorylation of ERK1/2, 30 μM), or p38 (SB203580, SB, 10 μM) enzyme activity prior to adding TNF-α. We confirmed that TNF-α significantly increased nuclear translocation of the p65 NF-ĸB subunit compared to the vehicle (Figure 3A,B). Blockage of CSE by pretreating the cells with PAG or BCA significantly suppressed TNF-α-stimulated NF-ĸB activation, indicating that the process was regulated by the CSE/H_2_S axis. Interestingly, there was a marked difference in the level of this suppressing effect between the two CSE inhibitors (Figure 3B) that could be explained by their different nature of CSE inhibition (discussed later). In addition, inhibition of ERK1/2 or p38 did not alter TNF-α-mediated NF-ĸB activation, suggesting that TNF-α stimulates NF-ĸB signaling independently from the activation of these MAPK pathways. We also examined the levels of p65-NF-ĸB in the nuclear extracts from HCECs after treating these cells with TNF-α and/or PAG. Nuclear fractionation followed by Western blotting and densitometric analysis revealed that CSE inhibition did not affect baseline levels of nuclear p65-NF-ĸB but reduced the increase in nuclear p65-NF-ĸB levels after TNF-α stimulus (Figure 3C). These results verified the regulatory effect of CSE on TNF-α-induced NF-ĸB activation.

### 3.4. CSE Inhibition Blocks TNF-α-Mediated Transepithelial Permeability Increase Independently of MAPK Activity

**Figure 4:**
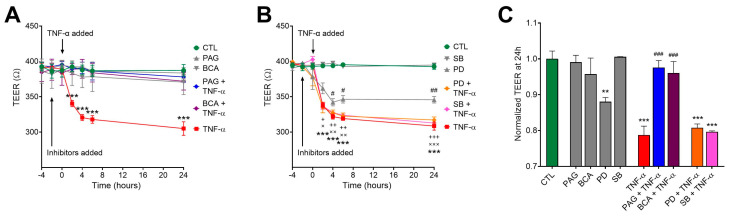
CSE promotes TNF-α-induced transepithelial permeability increase. HCECs were pretreated with inhibitors PAG, BCA (both 3 mM), PD98059 (PD, 30 μM), SB203580 (SB, 10 μM), or vehicle for 1 h, then treated with TNF-α (10 ng/mL) or vehicle for 30 min. Control cells (CTL) received the vehicle both times. Transepithelial electrical resistance (TEER) in confluent monolayers was measured for 24 h after treatments. Lower TEER values correlate with increased permeability. (**A**,**B**) TNF-α (10 ng/mL) significantly loosened up the barrier monolayer in 2 h and further increased barrier permeability until the 24 h endpoint. CSE inhibitors PAG and BCA had no effect alone, but they reverted TNF-α-induced reduction in TEER (**A**). The ERK1/2 inhibitor PD alone reduced TEER compared to control (CTL), but neither PD nor the p38 inhibitor SB altered the effect of TNF-α on TEER (**B**). (**C**) Then, 24 h after treatment, TNF-α induced a significant drop in the TEER value that was completely abolished by PAG and BCA but remained unaffected by PD or SB. Data are shown as mean ± SEM of *n* ≥ 3 independent experiments. ^+^ and ^x^, *p* ≤ 0.05, **, ^++^, and ^xx^, *p* ≤ 0.01, or ***, ^+++^, and ^xxx^, *p* ≤ 0.001, significantly different from the CTL group; ^#^ *p* ≤ 0.05, ^##^ *p* ≤ 0.01 or ^###^ *p* ≤ 0.001, significantly different from the TNF-α group: two-way ANOVA followed by Tukey’s post hoc test.

The process of intestinal epithelial restitution initiates with the complex rearrangement of barrier junctions and an increase in transepithelial permeability. In a previous study, we found that CSE activity was required for TNF-α regulation of transepithelial permeability [41]. To further investigate the regulatory role of the CSE/H_2_S pathway in colonic epithelial barrier function in response to TNF-α, HCECs were cultured until they formed tight monolayers. We measured electrical resistance across the epithelial monolayer (transepithelial electrical resistance (TEER)) to assess the integrity and permeability of the monolayer barrier (lower TEER values correlate with increased permeability). Our earlier studies showed that an intact, confluent monolayer of HCECs elicited approximately 400 Ohms (W) of TEER. After reaching confluency, we replaced the culture media with starvation media (containing 1% BSA) to maximize the biological responses to treatments. We monitored TEER over a 24-h-long period. Exposing the cells to TNF-α at different concentrations (1, 5, and 10 ng/mL) exerted a significant epithelial barrier disruption in a dose-dependent fashion. Selecting a 10 ng/mL concentration of TNF-α for further experiments, we found that CSE inhibition by PAG or BCA reversed the TNF-α-induced epithelial permeability (Figure 4A,C). In these experiments, TNF-α and the inhibitors were applied at both (luminal and basolateral) sides of the transwell system. Pretreating the cultures with CSE inhibitors alone had no effect on TEER compared to the control group. Blocking ERK1/2 signaling by adding PD alone significantly lowered the TEER of the monolayer, and the effect was more aggravated in the presence of TNF-α stimulus (Figure 4B,C). In contrast, inhibiting p38 alone with SB had no impact on barrier function. Adding PD or SB to TNF-α challenge did not significantly alter its effect. All these data suggest that TNF-α-mediated regulation of transepithelial permeability in the colon epithelial cells requires intact CSE and ERK1/2 activity, but TNF-α signaling overrides the need for ERK1/2 activation.

### 3.5. CSE Activity Stimulates TNF-α-Mediated Wound Healing

After the epithelial monolayer loosens up in response to injury, the cells proliferate and migrate to the wound to initiate tissue repair and restoration of mucosa integrity. TNF-α is a key regulator of these wound-healing processes (as well as inflammatory responses) through several different signaling cascades.

**Figure 5:**
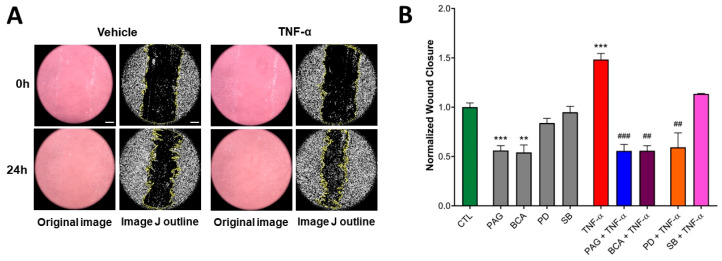
CSE activity is indispensable for basal and TNF-α-mediated HCEC wound healing. Wound-healing capacity of HCECs was assessed by the wound scratch assay using TNF-α (10 ng/mL) or mock (vehicle) stimulus after a 1 h pretreatment with inhibitors PAG, BCA (both 3 mM), PD98059 (PD, 30 μM), SB203580 (SB, 10 μM), or vehicle. Control cells (CTL) received the vehicle both times. The wound closure ratio was determined by measuring the wound area at different time points and normalizing it to the initial wound size using ImageJ software. (**A**) Representative microscopic images (all taken at 100× magnification) of HCEC cultures during the wound scratch assay and (**B**) normalized wound closure data (a higher wound closure value corresponds to a smaller remaining wound area) at 24 h after treatment revealed that TNF-α increased the wound closure capacity of HCECs. The inhibition of CSE by PAG or BCA significantly decreased basal wound closure. TNF-α-stimulated wound closure was suppressed by pretreating the cells with PAG, BCA, or PD. Data are shown as mean ± SEM of *n* ≥ 3 independent experiments. ** *p* ≤ 0.01 or *** *p* ≤ 0.001, significantly different from the CTL group; ^##^ *p* ≤ 0.01 or ^###^ *p* ≤ 0.001, significantly different from the TNF-α group: two-way ANOVA followed by Tukey’s post hoc test and unpaired, nonparametric Mann–Whitney test. Scale bars indicate 100 μm.

To investigate the role of CSE in intestinal restitution, we used a 2D wound scratch assay that allows the cells to respond to various stimuli, including the administration of TNF-α (10 ng/mL). The addition of TNF-α significantly stimulated the basal wound closure ability of HCECs over 24 h (Figure 5A,B). Pretreating the cells with the CSE inhibitors PAG or BCA (3 mM) blocked basal migratory responses and suppressed the stimulatory effect of TNF-α (Figure 5B). On the other hand, blocking ERK or p38 MAPK by adding PD or SB alone did not alter basal HCEC wound closure. However, the inhibition of ERK (but not p38) significantly suppressed TNF-α-stimulated wound closure (Figure 5B). These data suggest that TNF-α-mediated colon epithelial wound healing requires CSE/H_2_S activity and ERK1/2 activation.

### 3.6. Colonoid Cultures Confirm CSE Regulation of TNF-α Signaling Identified in HCECs

**Figure 6:**
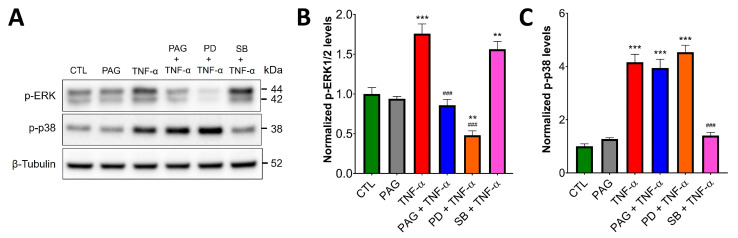
In colonoid cultures, CSE regulates TNF-α-induced ERK1/2, but not p38 signaling. Healthy human colon-derived organoids (colonoids) were pretreated with the specific CSE inhibitor PAG (3 mM), ERK1/2 inhibitor PD98059 (PD, 30 μM), p38 inhibitor SB203580 (SB, 10 μM), or vehicle control for 2 h, then treated with TNF-α (10 ng/mL, 30 min) or mock-treated (vehicle). Control cells (CTL) received the vehicle both times. ERK1/2 and p38 activation were analyzed by Western blotting using antibodies against phosphorylated ERK1/2 and p38, respectively. (**A**) Representative Western blot images and (**B**,**C**) densitometric analyses of *n* ≥ 3 independent experiments showed that TNF-α induced ERK1/2 and p38 activation. TNF-α-mediated ERK1/2 activation was blocked by PAG and PD, but none of these inhibitors affected p-p38 levels. SB inhibited p38 activation but did not affect TNF-alpha-induced ERK1/2 phosphorylation. ** *p* ≤ 0.01 or *** *p* ≤ 0.001, significantly different from the CTL group; ^###^ *p* ≤ 0.001, significantly different from the TNF-α group: one-way ANOVA followed by Dunnett’s post hoc test.

To verify our key findings about the effects of CSE inhibition on TNF-α signaling obtained from the HCEC model cell line, we used healthy human colon-derived organoids (colonoids) in similar experimental settings. Colonoids were pretreated with the specific CSE inhibitor PAG (3 mM), ERK1/2 inhibitor PD (30 μM), or vehicle control for 2 h, then treated with TNF-α (10 ng/mL, 30 min) or mock-treated (Figure 6). Western blotting and densitometric analysis revealed a virtually identical activation pattern for ERK1/2 and p38 MAPK to the one found in HCECs (Figure 2). TNF-α treatment increased the activation level of both ERK1/2 and p38, and TNF-α-mediated ERK1/2 (but not p38) activation was blocked by PAG and PD. These data suggest the relevance of using a simple, diploid cell line model to elucidate interactions between CSE and TNF-α signaling in human colon epithelium during injury response.

## 4. Discussion

In the past two decades, the role of the three major endogenous H_2_S-producing enzymes, CSE, CBS, and 3-MST, in maintaining homeostasis, attenuating inflammation, and promoting wound healing in the colonic mucosa has been abundantly established [35,39,42,53,54]. During approximately the same timeframe, anti-TNF-α agents have revolutionized the treatment of IBD, effectively reducing inflammation and supporting mucosal healing, but over 50% of patients never responded to this therapy, or the responses were not durable [19,55]. The fundamental reasons for the lack of responses to anti-TNF-α therapy are currently unclear; therefore, the regulatory processes of TNF-α signaling in the colonic mucosa under homeostatic and inflammatory conditions are being extensively investigated [17,55].

**Figure 7:**
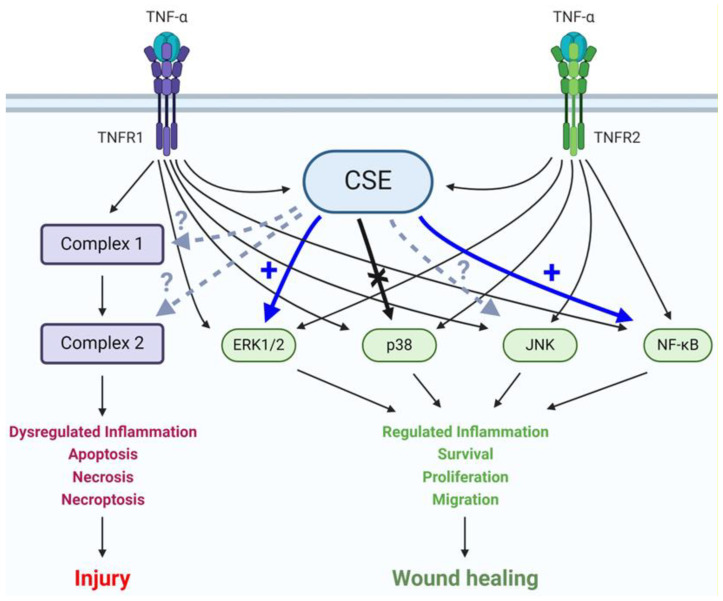
CSE activity regulates TNF-α signaling in colonocytes. A schematic summary of interac-tions between CSE activity and TNF-α signaling pathways in colonic epithelial cells. Colonocytes express both TNF-α receptors, TNFR1 and 2. Engagement of these receptors by TNF-α leads to a complex network of signaling pathways with different biological outcomes depending on the acti-vation status of key effector molecules and complexes. CSE promotes TNF-α-mediated ERK1/2 and NF-κB activation but does not affect p38 phosphorylation. The potential interactions of CSE with JNK, Complex 1 and 2, are currently unknown. Continuous thin arrow: known activation, continu-ous thick blue arrow: newly discovered promotion of activation, continuous thick black arrow: newly proven lack of interaction, and dashed arrow: interaction status unknown.

In an earlier study, we found significantly downregulated CSE expression in colonic mucosal biopsy specimens from UC patients and showed that the action of CSE promoted normal human colon epithelial cell migration, proliferation, and transepithelial permeability [41]. These results raised the possibility of a potential interaction between CSE- and TNF-α-mediated signaling in the colonic epithelium. In the present study, we aimed to explore this interaction using normal colonocytes. First, we demonstrated that TNF-α challenge stimulated CSE expression and CSE-derived H_2_S production in HCEC cultures. Then, we showed that simultaneously, CSE promoted TNF-α-induced ERK1/2 and NF-ĸB activation but did not affect p38 MAPK phosphorylation (Figure 7). Most importantly, we found that intact CSE activity was indispensable for TNF-α-mediated transepithelial permeability increase and wound healing in vitro. Key results obtained from HCEC cultures were also confirmed in an organoid model of the healthy human colon, validating their biological relevance.

TNF-α signaling has been shown to increase cellular H_2_S production via upregulating CSE expression in several different cell types, including adipocytes [56], chondrocytes [57], peritoneal macrophages [58], colonic muscle cells [59], and hepatocytes [58]; however, this mechanism has never been tested in colonocytes or any intestinal epithelial cell type. We were able to demonstrate a significant increase in the CSE protein level 24 h after TNF-α challenge in HCECs, but not at the 30 min time point. Nevertheless, 30 min after TNF-α treatment, we recorded an over 4-fold surge in the cellular H_2_S level, and based on our results from samples pretreated with CSE-specific inhibitors, over 90% of this increase was produced by CSE. While a TNF-α-induced surge in CSE activity in such a short timeframe has never been reported in any cell type, and most of the related literature focuses on the effects and mechanism of upregulated CSE transcription by TNF-α signaling [58], our data are more in line with studies by Wallace and his colleagues [36], who concluded that the great increase in the capacity of H_2_S production in rats with colitis was due to an increase in enzymatic activity rather than protein expression.

While there is no currently available data explaining the rapid, TNF-α-mediated CSE activity increase, several potential mechanisms could explain this phenomenon. Interestingly, four potential phosphorylation sites have been identified in CSE (Tyr60, Tyr114, Ser282, and Ser377), out of which phosphorylation of Tyr60, Tyr114, and Ser377 abrogates H_2_S production [60,61], but the function of Ser282 has not been investigated. Whether the phosphorylation of this amino acid by one of the active kinases involved in the TNF-α signaling cascade or any other posttranslational modification of CSE is responsible for TNF-α-induced early H_2_S response needs to be further explored. Another possibility is that TNF-α signaling activates CSE indirectly. A potential example could be via changes in the intracellular Ca^2+^ levels. CSE is physiologically activated by the calcium (Ca^2+^)/calmodulin complex but not by either substance alone [62]. TNF-α increased the intercellular free Ca^2+^ concentration by stimulating the release of Ca^2+^ from intracellular stores in rat cardiomyocytes [63], but whether this mechanism exists in colonocytes and its potential role in TNF-α/CSE interactions are currently unknown. Clearly, more mechanistic research will be needed to elucidate the specific molecular interactions explaining the kinetics of CSE function and overall H_2_S production after TNF-α stimulation of the colon epithelium.

The role and importance of the various metabolic pathways involved in endogenous H_2_S production in normal colonocytes are currently under debate [32,35,44]. To complicate matters, it seems likely that under different conditions, different enzymes could become the dominant source of H_2_S, warranting the re-examination of their relative impact in each experimental setting. To assess the contribution of CSE in HCECs in response to TNF-α challenge, we applied two CSE-specific inhibitors, PAG and BCA, and verified our key findings using siRNA-mediated silencing of CSE transcription. Minor disparities in our data obtained with PAG and BCA could be due to the different mechanisms of CSE inhibition by these two molecules. PAG exerts an irreversible, competitive inhibition by covalently binding to CSE and obstructing the enzymatically active site by steric hindrance [64]. BCA is an allosteric inhibitor occupying the PLP-binding site of CSE in a reversible manner, preventing enzymatic function that requires PLP as a cofactor [65,66]. Asimakopoulou et al. compared the selectivity and potency of several inhibitors for CSE and CBS, including PAG and BCA [67]. Although they reported that BCA was a more potent (IC50 14 ± 0.2 μM) inhibitor of CSE than PAG (IC50 40 ± 8 μM) using purified, recombinant enzyme, when we used a 3 mM concentration of each inhibitor in HCECs, their relative inhibitory effects varied depending on the assay. On the other hand, the same study showed that BCA also inhibited CBS when used above 1 mM, which could contribute to alterations in the H_2_S level and modulate cellular functions. While both inhibitors have been shown to inhibit several other enzymes, in terms of H_2_S production, PAG was found to be truly CSE-specific [67]. In this study, we found that both inhibitors blocked over 90% (93.9% by PAG and 96.7% by BCA) of all TNF-α-induced early H_2_S production, indicating that in colonocytes, CSE is responsible for most of the immediate increase in the endogenous H_2_S level after TNF-α stimulus, confirming earlier reports on animal models of colitis [38].

TNF-α plays an indispensable role in both physiological and pathological processes in the colon, initiating a complex network of signaling cascades that regulates mucosal homeostasis, inflammation, as well as epithelial injury and restoration [9,12,13], as schematically depicted in Figure 7. Both types of cellular TNF receptors (TNFR1 and TNFR2) are involved in intracellular signaling cascades, leading to both mucosal injury and wound healing [12,13]. Deciphering the regulation of these signaling pathways could provide the basis for improving current therapeutic strategies for inflammatory diseases of the colon. To this end, we evaluated whether the early, expression-independent surge in CSE activity interacted with TNF-α-induced signaling cascades and demonstrated that ERK1/2 and NF-κB activation were dependent on CSE function, while p38 MAPK activation was not. We also validated the biological relevance of key results in an organoid model derived from a healthy human colon. To our knowledge, this is the first report of CSE-regulated TNF-α signaling in intestinal epithelial cells, and our findings correlate well with recent reports studying other cell types [27,58,68]. The molecular mechanism of this regulation remains to be elucidated, but based on current literature, CSE-mediated *S*-sulfhydration of key signaling molecules, such as NF-κB, dual-specificity kinase MEK1, and others, seems highly probable. A comprehensive study of the human epithelial cell *S*-sulfhydrome, similar to a recent publication about the endothelial *S*-sulfhydrome [60], could provide major insights into regulative functions of H_2_S-producing enzymes and into the rapidly expanding literature about the role of H_2_S in intestinal health and disease.

To assess the potential biological effects of CSE regulation after TNF-α challenge in colonocytes, we used functional in vitro assays. The transepithelial electrical resistance (TEER) assay was developed to model the barrier function of a cellular monolayer [41]. Our results suggested that CSE function is essential for TNF-α-induced transepithelial permeability increase. While the permeability increase after TNF-α challenge is in line with current literature [14], the promoting role of CSE in this increase may seem counter-intuitive, since most available data suggest that endogenous H_2_S supports epithelial barrier integrity [30,39,69]. One probable explanation is that CSE exerts its barrier protective effects by promoting epithelial tissue restoration after injury, which requires the mobilization of epithelial cells, temporarily increasing barrier permeability by breaking up the monolayer. To test the impact of CSE activity on basal and TNF-α-mediated wound healing, we used an in vitro wound scratch assay. TNF-α has been recently recognized as an important factor in intestinal wound healing [12,13] and, accordingly, it significantly stimulated wound closure in our model. Importantly, CSE inhibition slowed down the basal wound-healing process by approximately 50% and completely abolished the impact of TNF-α. The most remarkable aspect of our results is that CSE activity was indispensable for any measurable effect of TNF-α in both functional assays, emphasizing the importance of ERK1/2 and NF-κB activation in these biological processes [26,28] and confirming the profound impact of CSE regulation on TNF-α-mediated colonic injury responses.

## 5. Conclusions

Taken together, our data demonstrated that CSE and TNF-α mutually regulated each other’s functions in colonocytes. TNF-α exerted an ‘early’ effect on CSE by stimulating its activity within minutes and a ‘late’ effect via upregulation of CSE expression, further increasing endogenous H_2_S production. In return, CSE activity promoted TNF-α-induced NF-κB and ERK1/2 activation but did not affect the p38 MAPK signaling pathway. As a result of these interactions, CSE activity not only strengthened TNF-α-mediated transepithelial permeability increase and wound healing but could be completely indispensable in these processes. Our results suggest that CSE activity is essential during intestinal injury response, and exploiting CSE regulation of intracellular signaling pathways could be beneficial as a therapeutic approach for diseases of the colon associated with impaired epithelial wound healing.

## Data Availability

All of the data is contained within the article and the Supplementary Materials.

## Supplementary Materials

The following supporting information can be downloaded at: https://www.mdpi.com/article/10.3390/antiox13091067/s1, Figure S1: SiRNA-mediated CSE silencing; Figure S2: Separation of cytoplasmic and nuclear extracts.
