# Supplementary material for: Cystathionine Gamma-Lyase Regulates TNF-α-Mediated Injury Response in Human Colonic Epithelial Cells and Colonoids
Source: Antioxidants (Basel). 2024 Aug 31;13(9):1067. doi: 10.3390/antiox13091067 (PMC11428476; doi:10.3390/antiox13091067)
Supplement: Supplementary file 1 [file antioxidants-13-01067-s001.zip › antioxidants-3103798-supplementary.pdf]

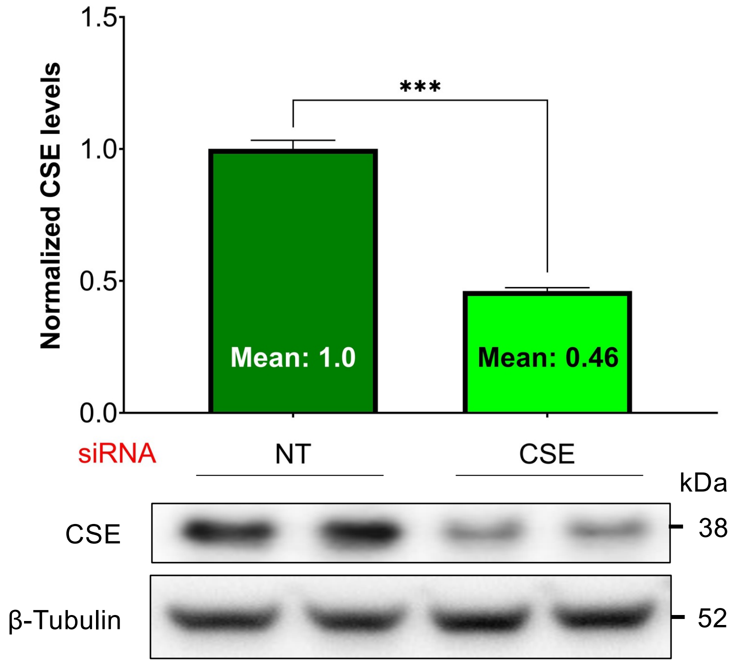

Figure S1. SiRNA-mediated CSE silencing. SiRNA oligonucleotides (CSE, 20 nM) were used for transient silencing of CSE in HCECs. For negative control, non-targeting siRNAs (NT, 20 nM) were used. Representative Western blot images are shown. Densitometric analyses of n = 3 independent experiments confirmed over 50% silencing efficiency. ***$p \leq 0.001$. Please note that under our experimental conditions (Methods) CSE runs at an observed molecular weight of 38 kDa, not at the predicted 42-44 kDa, as confirmed by siRNA silencing (Figure 2D).

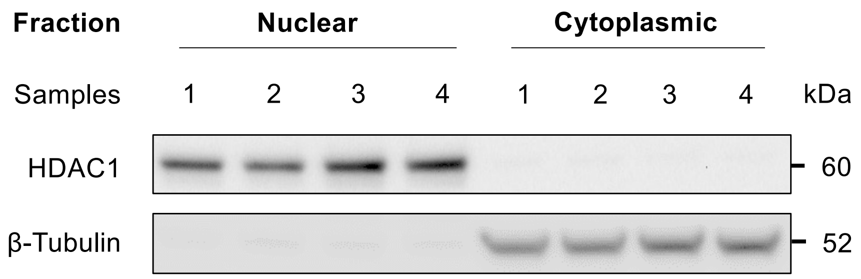

Figure S2. Separation of cytoplasmic and nuclear extracts. Cytoplasmic and nuclear fractionation of HCEC lysates was performed as described in the Methods section. Representative images of Western blotting using antibodies against cytoplasmic (β-tubulin) and nuclear (Histone deacetylase 1; HDAC1) housekeeping proteins are shown. Densitometric analysis confirmed that there was less than 1% cytoplasmic protein contamination in the nuclear fraction (data not shown).

Original, Full Length Western Blot Images for Figure 1.

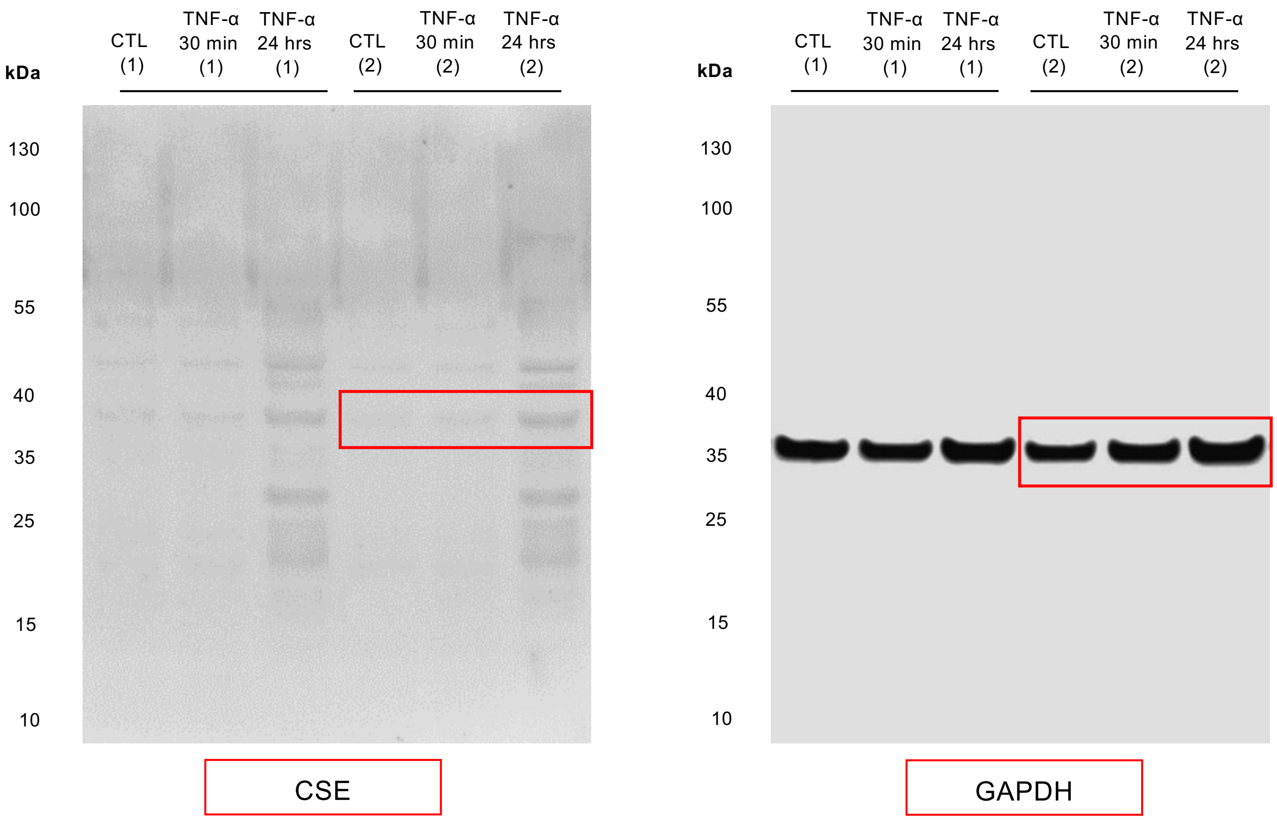

Please note that under our experimental conditions (Methods) CSE runs at an observed molecular weight of 38 kDa, not at the predicted 42-44 kDa, as confirmed by siRNA silencing (Figure 2D).

Original, Full Length Western Blot Images for Figure 2A.

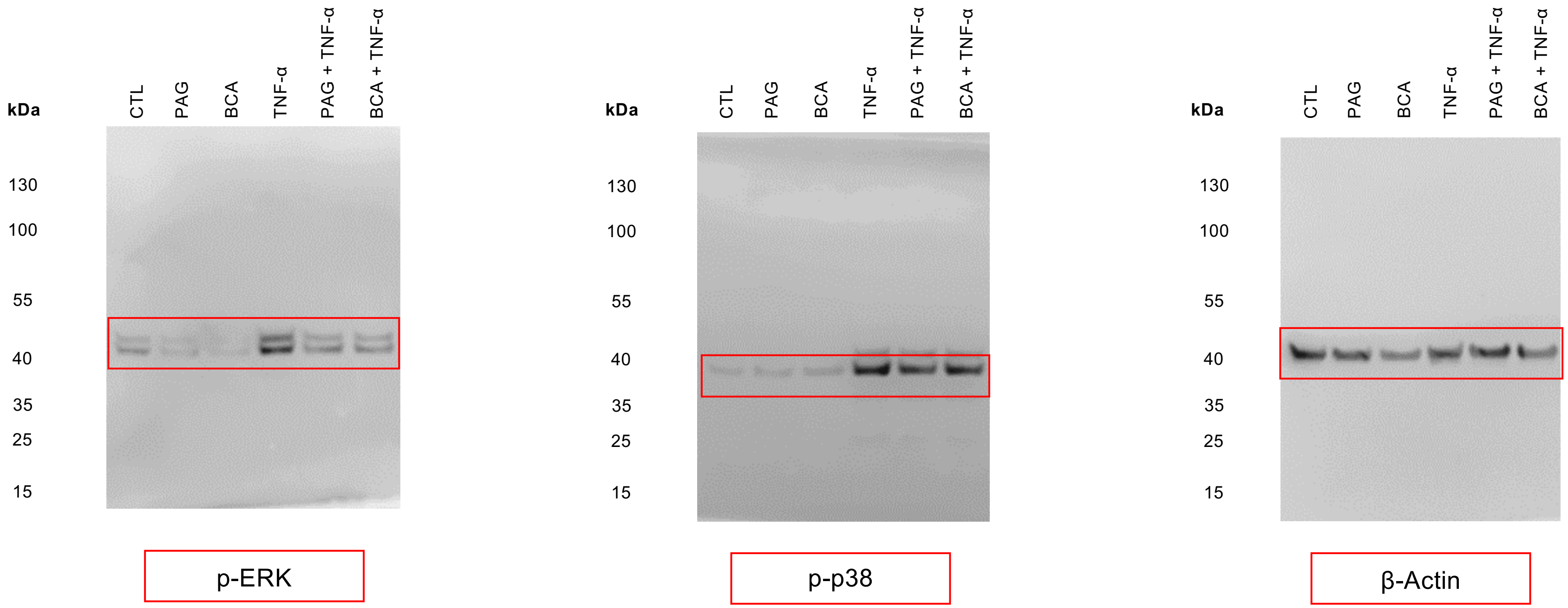

Original, Full Length Western Blot Images for Figure 2D.

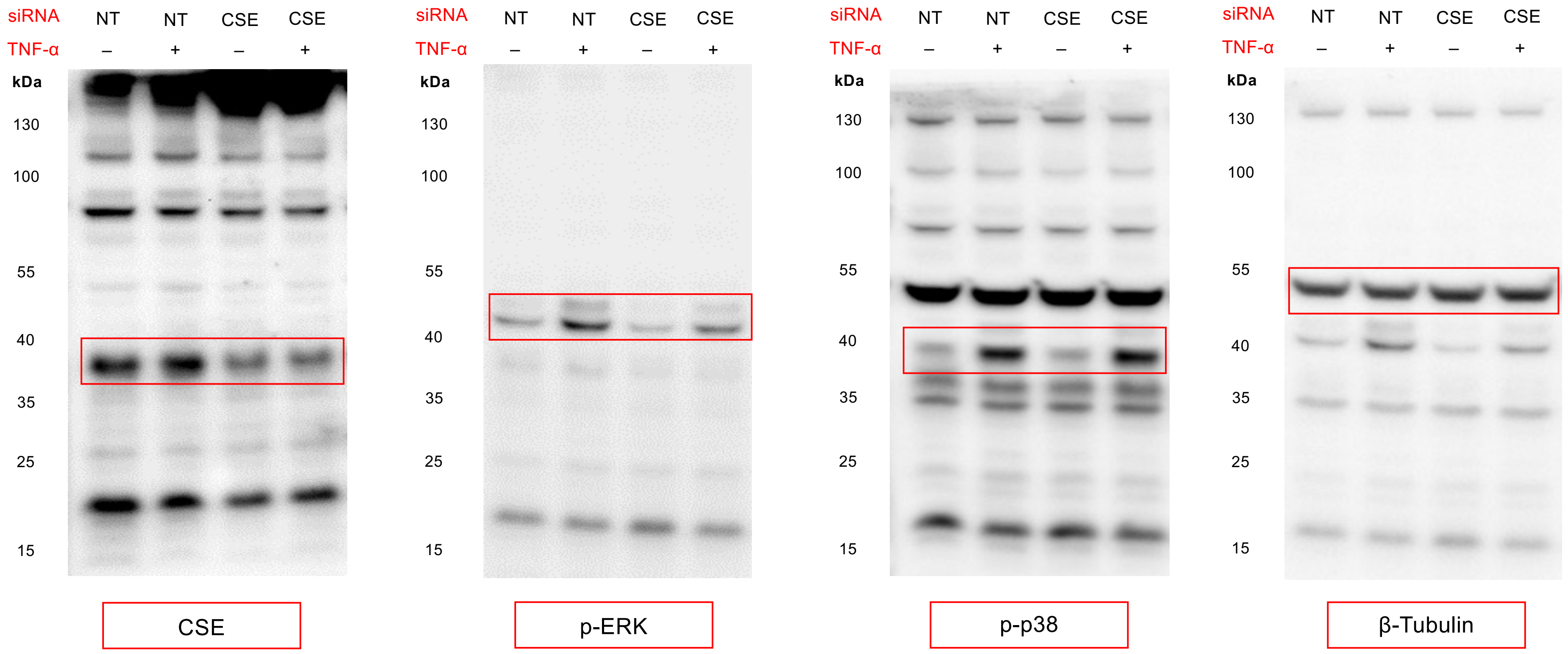

Please note that under our experimental conditions (Methods) CSE runs at an observed molecular weight of 38 kDa, not at the predicted 42-44 kDa, as confirmed by siRNA silencing. Antibodies were added to each blot in sequential order.

Original, Full Length Western Blot Images for Figure 3.

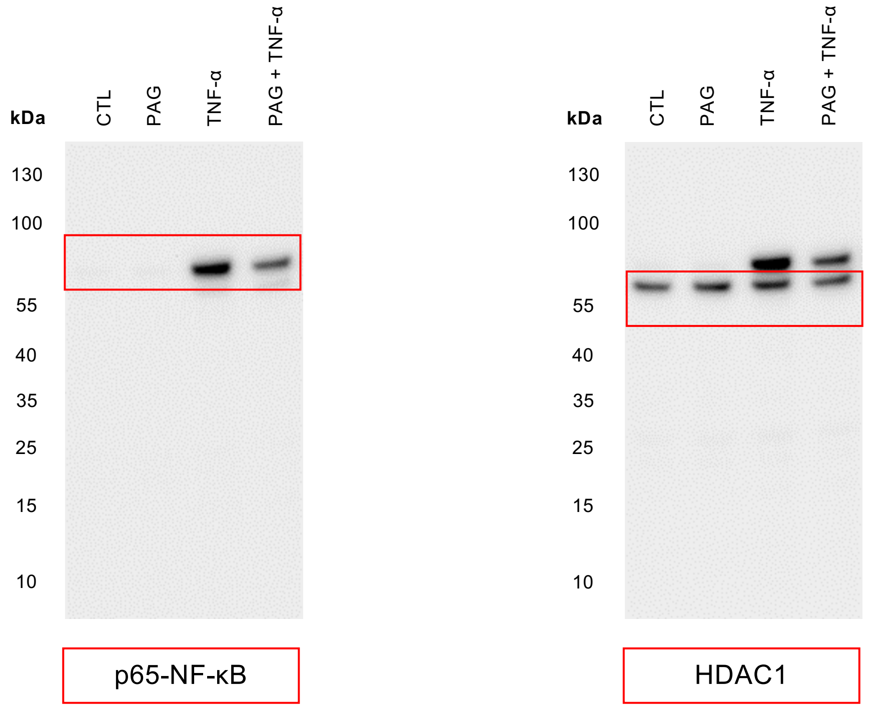

Please note that antibodies were added to each blot in sequential order.

Original, Full Length Western Blot Images for Figure 6.

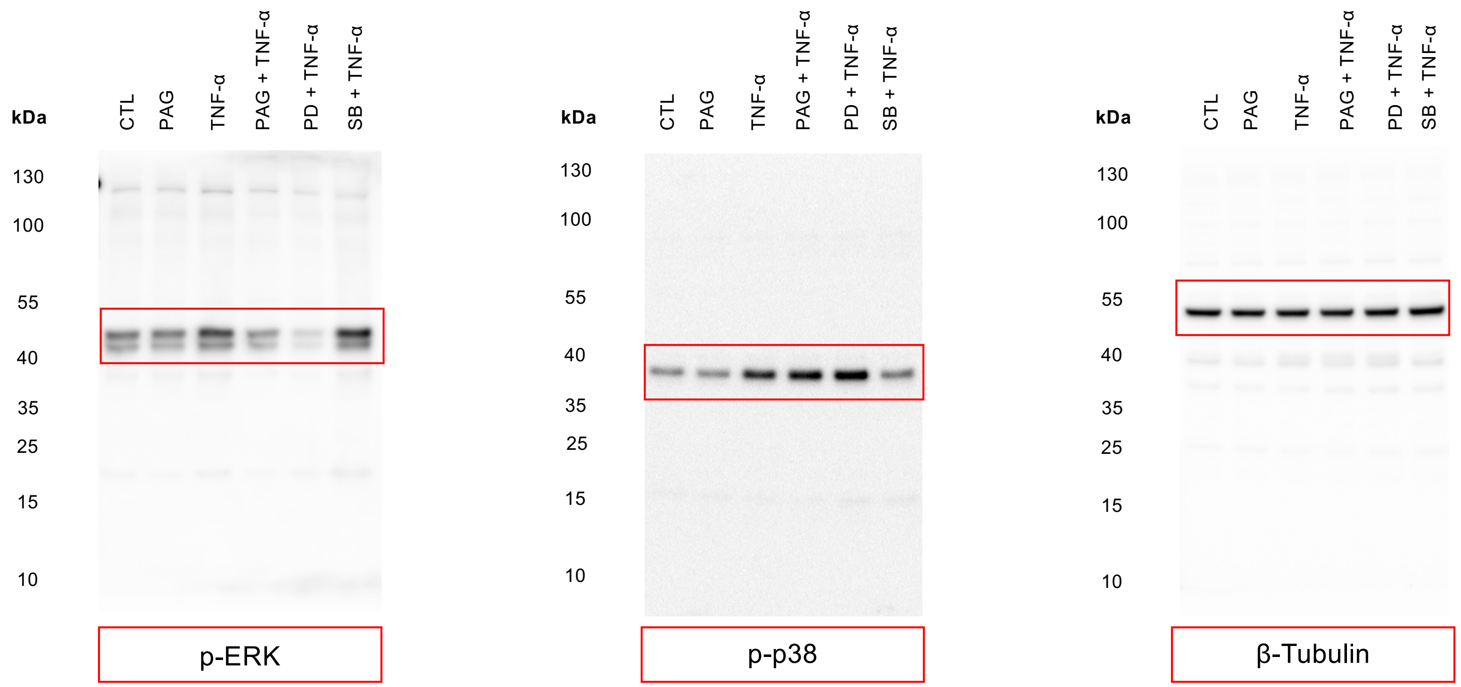

Original, Full Length Western Blot Images for Figure S1.

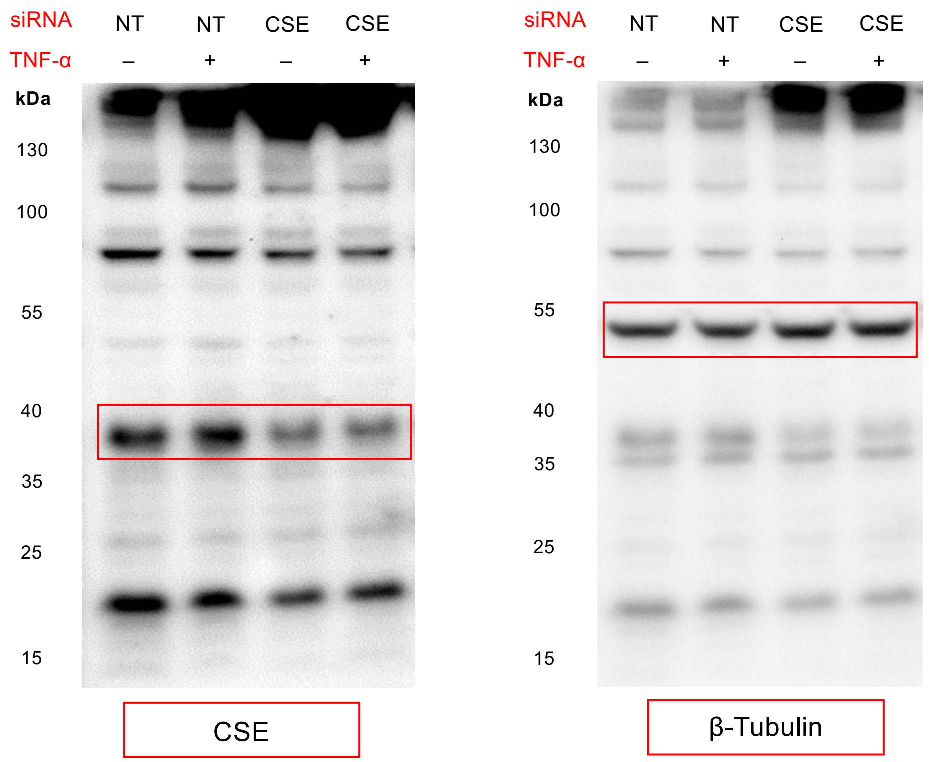

Please note that under our experimental conditions (Methods) CSE runs at an observed molecular weight of 38 kDa, not at the predicted 42-44 kDa, as confirmed by siRNA silencing (Figure 2D). Antibodies were added to each blot in sequential order.

Original, Full Length Western Blot Images for Figure S2.

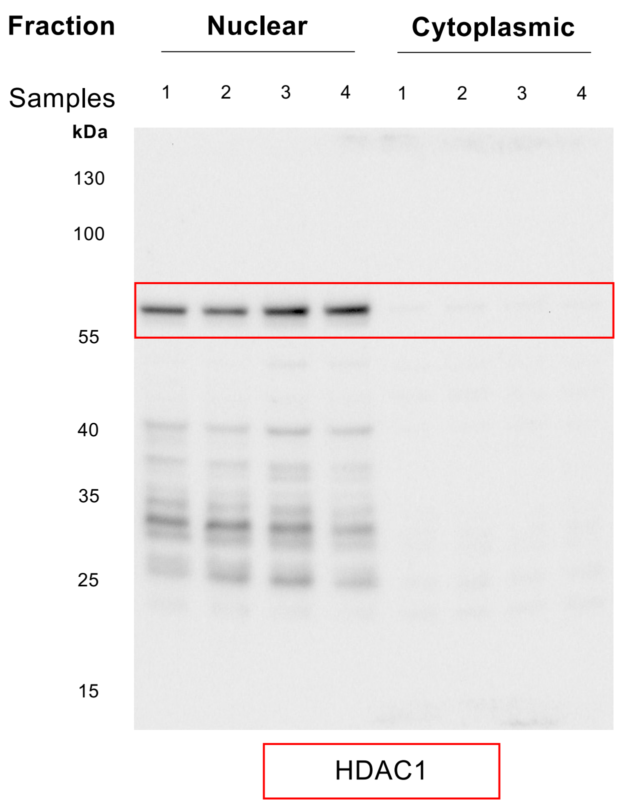

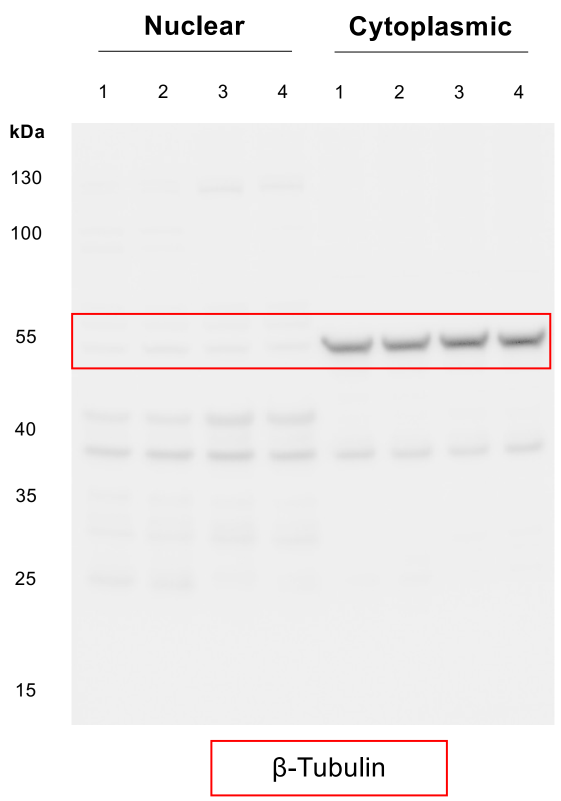

Please note that antibodies were added to the blot in sequential order (HDAC1 first, then β-Tubulin).
